# Outcomes of community-based nutrition support groups in Dioila Health District, Mali

**DOI:** 10.3389/fnut.2026.1753938

**Published:** 2026-01-21

**Authors:** Sulemana Seidu Dolo, Limkile Mpofu

**Affiliations:** 1 Ministère de la Santé et du Développement Social, Département Odontostomatologie, Bamako, Mali; 2 Department of Post Graduate Studies, Health Sciences, Africa Research University (ARU), Lusaka, Zambia; 3 Department of Psychology, College of Human Sciences, University of South Africa (UNISA), Pretoria, South Africa

**Keywords:** acute malnutrition recovery, caregiver participation, community-based nutrition interventions, infant and young child feeding (IYCF), Koulikoro Region, nutrition support groups (NSGs), rural Mali

## Abstract

**Background:**

Malnutrition remains a major public health concern in Mali, particularly in rural districts where food insecurity, limited dietary diversity, and inadequate infant and young child feeding (IYCF) practices persist. The Koulikoro Region continues to experience malnutrition levels above the national average. To strengthen community-level prevention and early detection, the Ministry of Health and partners implemented Nutrition Support Groups (NSGs), facilitated by relais communautaires. Despite their scale-up, evidence on NSG functionality and effectiveness in rural settings, including Dioila Health District, remains limited.

**Objective:**

To assess caregiver participation in NSGs, examine self-reported changes in feeding practices, and review recovery outcomes for moderate and severe acute malnutrition using routine health facility records.

**Methods:**

A cross-sectional study was conducted in Dioila District between August and September 2024. Caregiver knowledge and practices were assessed using a pre-tested questionnaire, while NSG attendance registers and health facility records provided data on participation and recovery. Descriptive statistics summarized variables, and chi-square and *t*-tests explored associations between NSG participation and caregiver outcomes.

**Results:**

A total of 227 caregivers from 37 villages participated in the study. NSG attendance was high (91%), and satisfaction with activities reached 78%. Frequent attendees demonstrated significantly higher nutrition knowledge scores (mean = 75.4 vs. 65.0; *t* = 5.23, *p* = 0.0002) and better feeding practices (*χ*^2^ = 15.67, *p* = 0.0001) compared with infrequent participants. Self-reported behavior changes included improved complementary feeding (42%), increased dietary diversity (28%), and enhanced hygiene practices (17%). Health facility records indicated strong recovery outcomes, with 99.86% for moderate acute malnutrition (MAM) and 97.86% for severe acute malnutrition (SAM). Reported challenges included limited funding, volunteer fatigue, and occasional community resistance.

**Conclusion:**

NSGs in Dioila Health District are effective community-based platforms for improving caregiver knowledge, promoting positive feeding practices, and supporting early detection of malnutrition. While facility-based recovery outcomes are strong, sustainability challenges remain. Strengthening supervision mechanisms, ensuring consistent support for community volunteers, and improving referral linkages with health facilities are critical to enhance the effectiveness and long-term sustainability of NSGs in rural Mali.

## Introduction

Malnutrition remains a significant public health challenge in Mali, particularly among children under the age of five (1). Both chronic and acute forms of undernutrition contribute substantially to child morbidity and mortality, undermine cognitive development, and reduce long-term human capital (2). National and international data indicate that chronic malnutrition (stunting) affects between 20% and 26% of children under five in Mali (3). Meanwhile, the prevalence of acute malnutrition remains high, with rates of global acute malnutrition (GAM) in certain regions and during lean seasons approaching or exceeding 10%, raising concerns about both the scale and severity of the problem (4).

The Koulikoro Region, where the Dioila Health District is located, is especially vulnerable. Rural districts such as Dioila face compounded structural and socioeconomic risk factors, including food insecurity during lean seasons, limited dietary diversity, low maternal literacy, and constrained access to preventive and curative health services (5). These factors are intertwined with suboptimal infant and young child feeding (IYCF) practices, further fueling undernutrition. Broader research in Mali has shown that low exclusive breastfeeding rates and poor complementary feeding practices are linked to entrenched social norms and community behavioral expectations (6). Climatic variability is increasingly recognized as a key determinant of undernutrition, as erratic precipitation and rising temperatures impact food production and household food access, thereby exacerbating nutritional risks in children (7).

To address these challenges, Mali’s Ministry of Health, together with international and non-governmental partners, has introduced community-based nutrition strategies, including Nutrition Support Groups (NSGs), locally referred to as *Groupes de Soutien aux Activités de Nutrition* (GSAN) (Ministère de la Santé et de l’Hygiène Publique, 2017). At the heart of this approach are “*relais communautaires*,*”* the community volunteers who facilitate peer-led gatherings, nutrition education, growth monitoring, screening for malnutrition, and referral to health facilities (8). NSGs serve multiple interrelated purposes, such as the following:

Behavior changes and caregiver empowerment: Peer support, cooking demonstrations, and regular meetings enable mothers to exchange knowledge and adopt improved IYCF practices (9).

Active screening and early detection: Relais communautaires conduct monthly anthropometric screening (e.g., mid-upper arm circumference) to identify children at risk of acute malnutrition (10).

Strengthening the continuum of care: NSGs bridge communities and health services, encouraging referral of severe cases and providing follow-up support. Evidence from southern Mali shows that revived NSGs increased screening and referrals, while moderate acute malnutrition cases decreased after community treatment was reintroduced (11).

Social mobilization and sustainability: Community ownership and local leadership enhance sustainability. Mali’s national protocol for integrated malnutrition care (PCIMA) explicitly identifies GSAN and relais as critical actors in community mobilization (Ministère de la Santé et de l’Hygiène Publique, 2017).

Despite the NSG promise, empirical evidence remains limited regarding their performance in rural settings, such as Dioila. Key questions persist regarding caregiver participation, mechanisms of behavior change, nutritional outcomes, and sustainability. In Dioila, where NSGs have been implemented for several years, a focused evaluation is especially valuable. This study examines caregiver participation, reported knowledge, and feeding practices, as well as child nutritional outcomes, thereby contributing to the growing body of evidence on community-led nutrition programming in Mali.

Objective: The primary objective is to evaluate caregiver nutrition knowledge, feeding practices, and participation in NSG activities, and to describe the recovery outcomes of Severe Acute Malnutrition (SAM) and Moderate Acute Malnutrition (MAM) in the Dioila Health District.

## Materials and methods

### Study design

This study employed a cross-sectional quantitative design to assess caregiver knowledge, feeding practices, and participation in Nutrition Support Group (NSG) activities within the Dioila Health District. Data were collected through structured, interviewer-administered surveys targeting caregivers of children under the age of five. The survey instrument captured sociodemographic characteristics, NSG attendance patterns, and key indicators of infant and young child feeding (IYCF) practices. While health facility records were reviewed to extract recovery outcomes for acute malnutrition, no qualitative methods were applied in this phase. Cross-sectional designs are widely used in nutrition research to generate population-level insights and explore associations between exposure and outcome variables at a single point in time (12, 13).

### Study setting

The research was conducted in the Dioila Health District, located in the Koulikoro Region of Mali. Dioila is a predominantly rural district characterized by subsistence agriculture, seasonal food insecurity, and limited access to diversified diets and health services. These contextual factors make it an appropriate setting for evaluating the outcomes of community-based Nutrition Support Groups (NSGs).

### Participants, sampling strategy, and sample size

The study population comprised caregivers of children under 5 years of age who were actively engaged in NSG activities. Sampling was conducted using systematic random sampling proportional to village size. A list of NSG members in each village served as the sampling frame; within each selected village every kᵗʰ member was selected where k varied by village according to the list length.

The minimum required sample size was calculated using the standard single-proportion formula:


n=Z2p(1−p)d2

were:

*Z* = 1.96 for 95% confidence, *p* = 0.60 (district estimate of optimal IYCF practice adoption), and *d* = 0.05.

This gives *n* ≈ 369. Because the unit of data collection was the village (cluster of ~10 active NSG members), the planned survey targeted 37 villages (369/10 ≈ 37). Due to logistical constraints and participant availability, 227 caregivers were ultimately enrolled. We acknowledge the smaller sample size as a limitation and discuss its potential impact on statistical power.

The unit of sampling for this study was the village. Each village contained at least one GSAN, with an average of 10 active members per GSAN. The number of villages to be surveyed corresponded to the total number of agents to investigate divided by the average number of agents per GSAN, which was 369/10. Therefore, a total of 37 villages were surveyed. However, due to logistical constraints and participant availability, a total of 227 participants were actually sampled using systematic random sampling across selected villages.

### Inclusion and exclusion criteria

#### Inclusion criteria

Mothers or primary caregivers of children aged 0–59 months, Active NSG membership for ≥6 months prior to data collection, Residency in the selected village for ≥1 year, and provided informed consent.

#### Exclusion criteria

Recent NSG members (<6 months), Temporary residents, Individuals unable to participate due to illness or communication barriers.

### Data collection

Data were collected using three complementary sources:

Structured Questionnaires: Caregivers completed interviewer-administered questionnaires assessing knowledge, attitudes, and practices related to infant and young child feeding (IYCF). Structured tools have been validated for measuring IYCF practices in low-resource settings (13). Although the tool primarily generated quantitative data, selected open-ended questions were included to capture contextual information on NSG recovery outcomes for acute malnutrition, which informed interpretation of the quantitative findings.

To give an example, based on 20 questions: the responses were scored as follows- Correct response was scored 1, while incorrect or “do not know” responses were scored 0, yielding a total possible knowledge score ranging from 0 to 20. Total scores were converted into percentages and categorized as follows: Low (<50%), Moderate (50%–74%), and High (≥75%).

Feeding practices, on the other hand, were evaluated using the standard WHO Infant and Young Child Feeding (IYCF) (14) indicators, including exclusive breastfeeding for infants 0–5 months, minimum meal frequency, and minimum dietary diversity for children aged 6–23 months. For the composite feeding-practice score, each recommended practice achieved was assigned 1 point. The points were summed and classified into Low, Moderate, and High adherence using tertiles based on the distribution of practice scores.

All scoring tools, cutoffs, and indicators were pre-tested and refined during the pilot phase to improve clarity and ensure cultural appropriateness.

Attendance Registers and NSG Activity Logs: Records maintained by relais communautaires were reviewed to document caregiver participation frequency, consistency, and type of engagement, for example, cooking demonstrations, peer discussions, and home visits.

Anthropometric data: Health facility registers were reviewed to extract child-level anthropometric indicators, including mid-upper arm circumference (MUAC) and weight-for-height *z*-scores. These measures are standard for identifying acute malnutrition and monitoring recovery outcomes (15).

### Data analysis and management

Data were entered into statistical software and analyzed using descriptive statistics to summarize caregiver characteristics, participation rates, and child nutritional outcomes. Chi-square tests were applied to compare IYCF knowledge and feeding practices between caregivers with high versus low NSG participation. We describe how we analysed and managed our data below:

Data entry and cleaning: Data were double entered into a secure database (Microsoft Excel) and validated; discrepancies were reconciled by reviewing original paper forms. Software: Analyses were performed using Stata v16 (Stata Corp).
Descriptive statistics: We calculated frequencies and percentages for categorical variables and means (with standard deviations) or medians (with interquartile ranges) for continuous variables.
Bivariate analysis: To test associations between NSG attendance (frequent vs. infrequent) and categorical outcomes (e.g., appropriate feeding practice) we used chi-square tests (or Fisher’s exact test where cell counts <5). To compare means of continuous outcomes (e.g., knowledge scores) between two groups, we used independent samples *t*-tests after checking for normality. Where distributions were non-normal, we used the Mann–Whitney *U* test. Reported *p*-values are two-tailed, and statistical significance was set at *p* < 0.05.
Multivariable analysis: To adjust for potential confounders (e.g., caregiver age, education, household socioeconomic status), we fitted logistic regression models for binary outcomes and linear regression for continuous outcomes. Variables were selected for multivariable models based on *a priori* knowledge and bivariate screening (*p* < 0.20). Model fit and multi-collinearity were assessed using standard diagnostics.
Sensitivity analyses: Where appropriate, sensitivity analyses were performed (for example, using alternate cut-offs for knowledge categories or excluding observations with missing key covariates).
Reporting: For all major findings, we report effect estimates (mean differences, odds ratios), 95% confidence intervals, and exact *p*-values. Any deviations from the planned analytic approach are described in the Results and Limitations.
Recovery data: Recovery outcomes for SAM and MAM were extracted from facility registers covering the 12-month period preceding the survey (August 1, 2023 – July 31, 2024).

For each case, we recorded the admission date, diagnosis (SAM/MAM), treatment modality (outpatient/inpatient), discharge outcome (recovered, died, defaulted, or transferred), and length of stay, where available. Registers did not include systematic post-discharge follow-up to detect relapse.

### Data quality and rigour

To ensure rigour, the following procedures were implemented: The questionnaire was developed in English, translated into the local language(s), and back-translated to ensure fidelity; The instrument was pilot tested on 20 caregivers and revised for clarity and cultural appropriateness; Data collectors received 3 days of training on interview techniques, informed consent procedures, and anthropometric measurement (when applicable); Supervisors conducted spot checks and re-interviews on approximately 10% of completed forms to verify data completeness and interviewer reliability; During analysis, range and consistency checks were performed; implausible or missing values were queried against the original forms and corrected or coded as missing; Internal consistency of multi-item scales was assessed using item-total correlations and Cronbach’s alpha. Items with poor performance were reviewed and removed if justified (the computed alpha is reported in the Results and Supplementary material).

### Ethics and participant protection

The study protocol was approved by the ARU Research and Ethics Committee (HSHDC/0208/2024) and Ministère de la Santé et de l’Hygiène Publique (MOH/ERB/2024/03/09). All participants provided written (or thumbprint) informed consent prior to interviews. Interview data were anonymized at collection by using unique study IDs; no personal identifiers were included in the analytic data set. Hard copies of consent forms were stored in locked cabinets, and electronic data were password-protected. Participation was voluntary, and participants could decline or withdraw at any time. Regarding referrals, respondents with children identified as malnourished were referred immediately to the nearest health facility according to local referral protocols.

## Results

### Participant characteristics

A total of 227 caregivers from 37 villages in the Dioila Health District participated in the study. Table 1 presents their sociodemographic characteristics, household composition, and participation in NSG activities. The majority of caregivers (56%) were aged 25–35 years. More than half (55%) reported having one child aged 0–59 months, while 45% had two or more. Participation in NSG activities was high, with 91% of respondents reporting attendance at least once during the 3 months preceding the survey. Overall satisfaction with NSG activities was also high, with 78% of caregivers reporting that they were satisfied or very satisfied.

**Table 1:** Sociodemographic characteristics, household composition, and participation and satisfaction with nutrition support group (NSG) activities among caregivers in the Dioila Health District.

Characteristic	Category	Frequency (*n*)	Percentage (%)
Age of caregiver (years)	18–24	45	19.8
	25–35	127	56.0
	36–45	40	17.6
	>45	15	6.6
Number of children aged 0–59 months	1	125	55.1
	≥2	102	44.9
NSG participation in past 3 months	Attended	206	90.7
	Did not attend	21	9.3
Satisfaction with NSG activities	Very satisfied	78	34.4
	Satisfied	98	43.2
	Neutral	31	13.7
	Dissatisfied	14	6.2
	Very dissatisfied	6	2.6

### Nutrition knowledge and feeding practices

Nutrition knowledge scores were categorized into three levels: low, moderate, and high. 57% of caregivers demonstrated moderate nutrition knowledge, while 26% had high knowledge. Feeding practice indicators varied across participants. Exclusive breastfeeding rates were higher among caregivers with high NSG participation, while meal frequency and dietary diversity showed moderate improvements across the sample (see Table 2).

**Table 2:** Feeding practice score calculation.

Indicator	Assessment question	Scoring
Exclusive breastfeeding	Child 0–5 months received only breast milk in the last 24 h	Yes = 1, No = 0
Minimum meal frequency	Child received age-appropriate meals in last 24 h	Yes = 1, No = 0
Minimum dietary diversity	Child consumed ≥4 food groups in last 24 h	Yes = 1, No = 0
Continued breastfeeding	Child 12–23 months still breastfed	Yes = 1, No = 0
Hygiene practice	Caregiver washed hands before feeding child	Yes = 1, No = 0

Feeding practice indicators and scoring.

Scores summed across items.

Categorized into: Low adherence (lowest tertile); Moderate adherence (middle tertile); High adherence (highest tertile).

### Self-reported behavior change

Following participation in NSG activities, 87.7% of caregivers reported adopting at least one improved nutrition or health-related practice. The most frequently reported changes included: Improved complementary feeding practices (42%), increased dietary diversity (28%), and Improved hygiene practices (17%).

Caregivers described strategies such as introducing locally available legumes and vegetables into children’s diets, preparing thicker porridge to improve nutrient density, and handwashing before food preparation. These reported practices reflect direct application of NSG demonstrations and peer discussions.

### Association between NSG participation and outcomes

#### NSG participation and nutrition knowledge

Inferential analysis revealed significant associations between NSG participation frequency and nutrition knowledge: Thus, A chi-square test showed a significant relationship between participation frequency and knowledge level (*χ*^2^ = 15.67, df = 1, *p* = 0.0001). Independent-samples t-tests revealed that caregivers with high NSG participation had significantly higher mean knowledge scores (mean = 75.4) compared with those with low participation (mean = 65.0; *t* = 5.23, *p* = 0.0002).

These findings demonstrate that regular NSG attendance was statistically associated with higher nutrition knowledge scores.

#### Recovery outcomes for acute malnutrition

**Figure 1:**
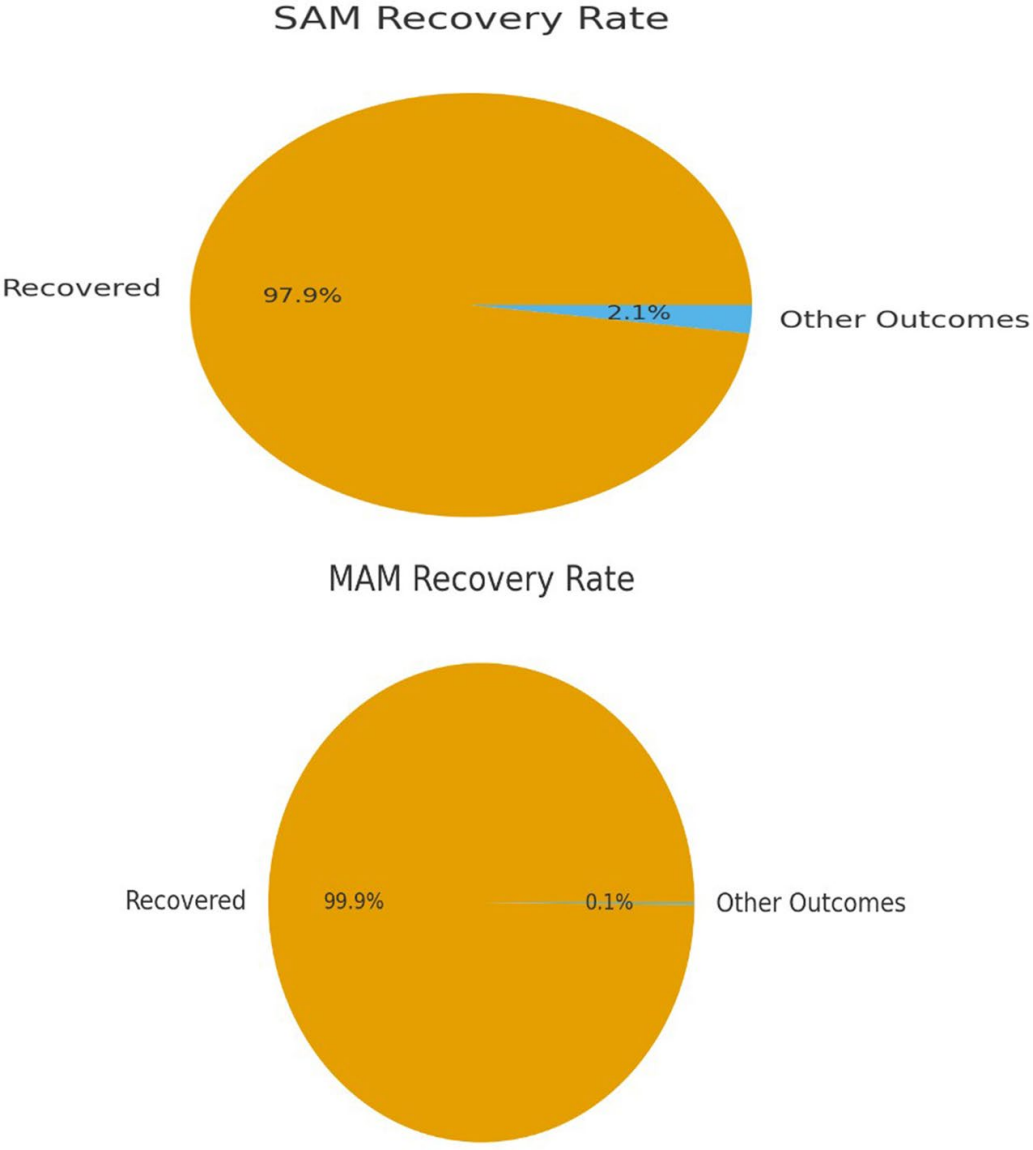
Recovery outcomes for SAM and MAM.

Recovery outcomes for acute malnutrition were derived from routine health facility registers covering the 12-month period from August 1, 2023, to July 31, 2024. Among cases with a recorded discharge outcome, the recovery rate was 99.86% for MAM and 97.86% for SAM (Figure 1). These figures reflect the proportion of children discharged as recovered among all cases with documented final outcomes during the reference period.

The registers provided information on admission diagnosis, treatment modality, and discharge status, but did not include systematic post-discharge follow-up. Therefore, relapse after recovery could not be assessed. The reported recovery rates represent facility-based discharge outcomes rather than sustained nutritional recovery over time.

### Challenges reported

Among 16 NSG facilitators and members who reported challenges, the most frequently cited barriers were: Limited funding (68.7%), Community resistance (25%), and Staff turnover (6.3%). These challenges were identified as factors that may affect the consistency, supervision, and long-term sustainability of NSG activities (see Table 3).

**Table 3:** Reported challenges to participation and support needs among Nutrition Support Group participants.

Challenges reported	Frequency (*n* = 16)	Percentage (%)
Limited funding	11	68.7
Community resistance	4	25.0
Staff turnover	1	6.3

### Summary of results

The findings highlight that there is High caregiver participation and satisfaction with NSGs. There were also statistically significant associations between NSG participation and improved nutrition knowledge. The tables further reflect a widespread self-reported adoption of improved feeding and hygiene practices, as well as strong recovery outcomes for acute malnutrition within community-based Management of Acute Malnutrition (CMAM) services, although not directly attributable to NSG participation. However, there are persistent challenges in funding, community acceptance, and staff retention that may affect sustainability.

## Discussion

This study examined the outcomes of community-based Nutrition Support Groups (NSGs) in the Dioila Health District, Mali, focusing on caregiver participation, nutrition knowledge, feeding practices, and recovery outcomes for acute malnutrition. The findings offer valuable insights into the role of NSGs in enhancing community-led nutrition programming in rural settings.

### Caregiver participation and satisfaction

High levels of caregiver participation (91%) and satisfaction (78%) underscore the acceptability of NSGs as a platform for nutrition education and peer support. Similar findings have been reported in community-based interventions across sub-Saharan Africa, where peer-led groups have achieved high engagement due to their cultural relevance and accessibility (5, 16). The strong participation rates in Dioila suggest that NSGs are well integrated into community structures, facilitated by relais communautaires who are locally chosen and trusted.

### Nutrition knowledge and feeding practices

The study demonstrated a significant association between NSG participation and higher nutrition knowledge scores. Caregivers who attended NSGs more frequently were more likely to report improved knowledge and practices, including exclusive breastfeeding, complementary feeding, and dietary diversity. These findings align with evidence from Ethiopia and Kenya, where community-based nutrition education significantly improved IYCF practices and caregiver knowledge (14, 17).

Self-reported behavior changes, such as introducing legumes and vegetables, preparing nutrient-dense porridge, and adopting hygiene practices, reflect practical strategies promoted during NSG sessions. These strategies align with recent studies highlighting the importance of locally available foods and culturally tailored demonstrations in enhancing dietary diversity (5, 13).

### Recovery outcomes for acute malnutrition

Facility-based recovery rates for MAM (99.86%) and SAM (97.86%) were high, consistent with global performance standards for community-based management of acute malnutrition (CMAM) programs (18). However, the absence of post-discharge follow-up data limits conclusions about sustained recovery. Importantly, the lack of individual-level linkage between NSG participation and facility outcomes prevents the direct attribution of recovery rates to NSGs. This gap highlights the need for integrated monitoring systems that connect community participation data with facility-based treatment outcomes, as recommended in recent evaluations of CMAM programs (19).

### Challenges to sustainability

Despite promising outcomes, NSG facilitators reported challenges including limited funding, community resistance, and staff turnover. These barriers mirror findings from other community nutrition programs in West Africa, where resource constraints and volunteer fatigue have threatened sustainability (20, 21). Addressing these challenges requires stronger integration of NSGs into district health systems, provision of incentives or recognition for volunteers, and continuous community sensitization to overcome resistance.

### Recommendations

Based on the study findings, several actions are recommended to strengthen Nutrition Support Groups (NSGs) and enhance child nutrition outcomes in the Dioila Health District.

### Improve data quality and health facility linkages

Strengthen health facility register management by standardizing documentation of admissions, defaulters, transfers, and outcomes.

Conduct regular audits and provide staff training to ensure the reliability of recovery data.

Establish clear referral pathways, joint review meetings, and bidirectional communication between NSGs and health facilities to support early detection and timely treatment of malnutrition.

### Sustain and expand community-based nutrition education

Scale up NSG coverage to reach previously unconnected villages.

Enhance program content with practical cooking demonstrations, home visits, and strategies to engage fathers and other family members.

Provide continuous training and supervision for facilitators to reinforce positive caregiver practices in nutrition and hygiene.

### Mobilize resources for program sustainability

Secure district-level funding and partnerships to support NSG transport, educational materials, facilitator incentives, and monitoring systems.

Address structural barriers such as financial constraints, staff turnover, and limited supervision to maintain program consistency and quality.

### Strengthen research and monitoring

Conduct longitudinal or mixed-methods studies to track caregivers and children over time, measure objective nutrition and health outcomes, and explore mechanisms through which NSGs influence knowledge, practices, and treatment-seeking behavior.

Use operational research to refine program delivery, improve supervision, and inform policy decisions.

### Implications for policy and practice

The results demonstrate that NSGs are effective platforms for enhancing caregiver knowledge and practices; however, gaps in sustainability and monitoring remain. The researchers highlight the following three key implications for Mali’s Ministry of Health:

1. Strengthen integration with health systems: Linking NSG participation data with facility records could enable more robust monitoring of outcomes.
2. Support relais communautaires: Incentives, supervision, and training are critical to sustain volunteer engagement.
3. Scale culturally tailored strategies: Cooking demonstrations and peer exchanges using locally available foods should be prioritized to enhance dietary diversity.

### Limitations

The study’s cross-sectional design limits the ability to make causal inferences. Recovery outcomes were derived from facility registers without individual-level linkage to NSG participation, and post-discharge relapse data were unavailable. Future longitudinal studies are needed to assess the sustained impacts of NSGs on child nutritional status.

## Conclusion

Community-based NSGs in Dioila Health District are associated with improved caregiver knowledge, feeding practices, and high satisfaction. While facility-based recovery outcomes for acute malnutrition are strong, sustainability challenges and monitoring gaps remain. Thus, strengthening NSGs as a cornerstone of rural nutrition interventions will require investment in volunteer support, integration with health systems, and culturally tailored approaches to behavior change. Incentivizing relais and reinforcing community ownership are critical for scaling.

## Data Availability

The raw data supporting the conclusions of this article will be made available by the authors, without undue reservation.
