# Supplementary material for: Outcomes of community-based nutrition support groups in Dioila Health District, Mali
Source: Front Nutr. 2026 Jan 21;13:1753938. doi: 10.3389/fnut.2026.1753938 (PMC12867911; doi:10.3389/fnut.2026.1753938)
Supplement: Supplementary file 1 [file Data_Sheet_1.pdf]

SUPPLEMENTARY ITEM

Table S1. Nutrition Knowledge Questions and Scoring Rubric

Domain	Question/Item	Scoring Method
Breastfeeding	Recommended duration of exclusive breastfeeding	Correct = 1, Incorrect/Don't know = 0
Breastfeeding	Time of initiation of breastfeeding after birth	Correct = 1, Incorrect/Don't know = 0
Breastfeeding	Need for water before 6 months of age	Correct = 1, Incorrect/Don't know = 0
Complementary feeding	Appropriate age to introduce complementary foods	Correct = 1, Incorrect/Don't know = 0
Complementary feeding	Minimum meal frequency (6–8 months)	Correct = 1, Incorrect/Don't know = 0
Complementary feeding	Minimum meal frequency (9–23 months)	Correct = 1, Incorrect/Don't know = 0
Complementary feeding	Importance of thick/energy-dense foods	Correct = 1, Incorrect/Don't know = 0
Dietary diversity	Ability to name ≥4 food groups	Correct = 1, Incorrect/Don't know = 0
Dietary diversity	Importance of animal-source foods	Correct = 1, Incorrect/Don't know = 0
Micronutriments	Importance of vitamin A–rich foods	Correct = 1, Incorrect/Don't know = 0
Hygiene	Handwashing before food preparation	Correct = 1, Incorrect/Don't know = 0
Hygiene	Handwashing before feeding a child	Correct = 1, Incorrect/Don't know = 0
Illness feeding	Continue feeding during illness	Correct = 1, Incorrect/Don't know = 0
Illness feeding	Give extra food after illness	Correct = 1, Incorrect/Don't know = 0
Growth monitoring	Purpose of MUAC measurement	Correct = 1, Incorrect/Don't know = 0
Growth monitoring	Meaning of red MUAC zone	Correct = 1, Incorrect/Don't know = 0
Malnutrition	Recognition of visible wasting	Correct = 1, Incorrect/Don't know = 0
Malnutrition	Recognition of bilateral oedema	Correct = 1, Incorrect/Don't know = 0

Domain	Question/Item	Scoring Method
Care-seeking	When to seek care for poor appetite	Correct = 1, Incorrect/Don't know = 0
Care-seeking	When to seek care for weight loss	Correct = 1, Incorrect/Don't know = 0

Knowledge Score Calculation

- Total possible score : **0–20**
- Scores converted to percentage
- Categories :

Low knowledge : < 50%

Moderate knowledge : 50–74%

High knowledge : ≥ 75%

Table S2. Feeding Practice Indicators and Scoring

Indicator	Assessment Question	Scoring
Exclusive breastfeeding	Child 0–5 months received only breast milk in the last 24 hours	Yes = 1, No = 0
Minimum meal frequency	Child received age-appropriate meals in last 24 hours	Yes = 1, No = 0
Minimum dietary diversity	Child consumed ≥4 food groups in last 24 hours	Yes = 1, No = 0
Continued breastfeeding	Child 12–23 months still breastfed	Yes = 1, No = 0
Hygiene practice	Caregiver washed hands before feeding child	Yes = 1, No = 0

Feeding Practice Score Calculation

- Scores summed across items
- Categorized into :

Low adherence (lowest tertile)

Moderate adherence (middle tertile)

High adherence (highest tertile)

New Table – NSG Activities

Table 3. Documented NSG Activities in Dioila District

Activity	Description	Frequency (typical)
Group education sessions	Peer-led discussions on IYCF, hygiene, and child care	Monthly
Cooking demonstrations	Preparation of nutritious complementary foods using local ingredients	Monthly/Quarterly
MUAC screening	Community-based MUAC measurement by relais communautaires	Monthly
Home visits	Follow-up of at-risk children and counselling of caregivers	As needed
Referral	Referral of SAM/MAM cases to health facilities	Ongoing
